# Complete surgical myocardial revascularization in patients with declined renal functions: 12-month outcomes

**DOI:** 10.1186/s12872-023-03507-1

**Published:** 2023-09-29

**Authors:** Ibrahim C. Kaya, Halil I. Bulut, Leilani Lopes, Merih Ozbayburtlu, Selim Kocaoglu

**Affiliations:** 1 Department of Cardiovascular Surgery, Eskisehir City Health Practice and Research Centers, Saglık Bilimleri Universitesi, Eskisehir, Turkey; 2 Cerrahpasa School of Medicine, Istanbul University-Cerrahpasa, Istanbul, Turkey; 3 Western University of Health Sciences College of Osteopathic Medicine of the Pacific-Northwest, Lebanon, OR USA; 4 Department of Cardiovascular Surgery, Eskisehir City Health Practice and Research Centers, Eskisehir, Turkey

**Keywords:** Coronary artery bypass grafting, Cardiopulmonary bypass, Kidney functions

## Abstract

**Introduction:**

This retrospective observational study aimed to evaluate the feasibility and effectiveness of complete revascularization coronary artery bypass grafting (CABG) in patients with multi-vessel disease (MVD)-CAD and declined renal functions, addressing the knowledge gap regarding optimal treatment strategies and outcomes in this specific patient population.

**Methods:**

Between 2020 and 2022, a total of 58 patients underwent on-pump coronary artery bypass grafting surgery for complete myocardial revascularization in this study. To assess overall survival, Kaplan–Meier with the log-rank test was conducted for statistical analysis.

**Results:**

The mean age of cohort was 60.7. The findings showed a high prevalence of medical conditions such as hypertension (50.0%), diabetes (50.0%), and anaemia (41.4%) among the participants. Intraoperatively, low cardiac output syndrome was reported in 5.2% of cases, while perioperative outcomes indicated a need for transfusions in 53.5% of cases and an in-hospital mortality rate of 3.4%. At the 12-month follow-up, no redo revascularization or renal replacement therapy was required, but cardiac mortality was 5.2% and all-cause mortality was 6.9%.

**Conclusions:**

The study concluded that complete revascularization is safe for these patients and highlights the potential benefits, emphasizing the need for further research in optimizing revascularization techniques for this population.

## Background

Impaired kidney function poses a significant challenge in coronary artery surgery, as it is associated with increased risks and adverse outcomes. Patients with renal impairment have a higher incidence of coronary artery disease (CAD) and face greater mortality risks. However, there is a lack of clear guidelines on the optimal treatment approach for CAD in this patient population, primarily due to their exclusion from major clinical trials and studies [1–6]. Furthermore, there is limited research investigating the outcomes of complete coronary revascularization in patients with impaired renal function, despite suggestions that it may yield better results compared to incomplete revascularization. This knowledge gap highlights the need for further investigation to determine the efficacy and benefits of complete revascularization in these patients [7, 8].

We conducted this retrospective observational study to assess the feasibility and effectiveness of complete revascularization CABG in patients with MVD- CAD and CKD. Our research aimed to evaluate the outcomes of CABG in this specific patient population and determine its potential benefits as a revascularization option. The study provides valuable insights into CAD management in individuals with impaired renal function and highlights the importance of complete revascularization via CABG for MVD-CAD patients with renal impairment.

## Methods

### Study design

This retrospective, single-centre study was conducted to report the 12-month outcomes of coronary bypass grafting specifically in patients with compromised renal function. The study aimed to provide valuable insights into the long-term outcomes of this surgical intervention in this particular patient population.

### Ethical considerations

After obtaining approval from the Health Science University Eskisehir Hospital Clinical Trials Ethics Committee, we proceeded to establish a de-identified database for the study. Adhering to the guidelines outlined by the committee and in accordance with ethical principles, we ensured the protection of patient privacy and confidentiality. The de-identification process involved removing any personally identifiable information from the dataset to maintain anonymity and uphold the highest standards of data security. By implementing these measures, we aimed to safeguard patient confidentiality while conducting a rigorous and ethically sound investigation. The Approval number is: " ESH/GOEK 2023/2”.

### Patients and inclusion criteria

Out of the 230 patients who underwent on-pump isolated coronary bypass grafting for complete revascularization between 2020 and 2022, 58 individuals met the study's inclusion criteria. The inclusion criteria for this study were as follows:

1. Underwent on-pump surgical complete revascularization for multivessel coronary artery disease, and
2. Met one or more of the following conditions:
  a Had an eGFR value below 90 ml/min/1.73m^2^, or
  b Had a diagnosis of CKD 3–5, or
  c Were undergoing CRRT treatment.

### Disease definitions

The diagnoses of the diseases selected as variables in this research were determined through a comprehensive examination of the patients' medical records and reports by the respective medical specialties. The diagnoses of conditions such as carotid disease (CD), acute myocardial infarction (AMI), previous myocardial infarction (PMI), structural heart disease (SHD), previous atrial fibrillation (AFib), type 2 diabetes mellitus (T2DM), chronic obstructive pulmonary disease (COPD), malignancy, anaemia, obesity, and severe renal impairment were established based on echocardiography reports, cardiology records, internal medicine records, pulmonology records, nephrology records, and consultations with relevant medical specialists. Additionally, consultations with nephrology and neurology specialists were sought for post-operative acute kidney injury (AKI) and stroke cases, and infectious disease specialists were consulted for the diagnosis of infections.

### Complete revascularization definition

In this study, complete revascularization was defined as the successful revascularization of all identified significant lesions. The determination of significant lesions was based on a diameter stenosis of 50% or more in vessels with a reference diameter of 2.0 mm or larger. However, it is important to highlight that in instances of stenosis in the posterior left ventricular artery (PLV) or posterior descending artery (PDA), bypass was performed to vessels with a reference diameter exceeding 1.5 mm. This approach ensured the comprehensive revascularization of all significant lesions, including those in the PLV and PDA, to optimize revascularization outcomes [8].

### Estimated glomerular filtration rate calculation

In this study, the glomerular filtration rate (GFR) was calculated using the MRDR4 formula. The equation used was as follows:

$$\text{eGFR} = 175 \times \text{(SCr)}^{\wedge}(\text{-}1.154) \times \text{(age)}^{\wedge}(\text{-}0.203) \times 0.742\ [\text{if female}] \times 1.212\ [\text{if Black}].$$

This formula took into account the serum creatinine level (SCr) and the age of the patients. Additionally, adjustments were made based on gender (0.742 for females) and race (1.212 for individuals of Black ethnicity). The calculated eGFR values provided an estimation of the patients' glomerular filtration rate, which was used as a measure of their renal function in the study [9].

#### Definition of declined renal functions

In this study, declined renal function was defined based on an estimated Glomerular Filtration Rate (eGFR) of less than 90. Patients with an eGFR of less than 60 and classified as having CKD level 3–5 were considered to have severe impairment of renal function [10]. Furthermore, three patients in the study were currently undergoing continuous renal replacement therapy with an eGFR less than 45.

### Procedures of renal protection

#### Conventional ultrafiltration

During the study, the group employed conventional ultrafiltration (CUF) as a technique from the rewarming stage until the completion of cardiopulmonary bypass (CPB). For modified ultrafiltration, a simplified arteriovenous technique was utilized. This technique involved the use of specialized equipment, including a 0.25-inch tubing line and a polysulfone hemofilter. To facilitate the process, a three-way connector was employed at the free end to connect both the inlet and outlet of the hemofilter to the 0.25-inch tubing [11].

In the case of conventional ultrafiltration (CUF), the inlet tubing of the hemofilter was connected to the arterial line, originating from the 40-μm arterial line filter. On the other hand, the outlet tubing of the hemofilter was connected to the venous reservoir through a three-way connector. This setup allowed for the effective removal of excess fluid and solutes during the bypass procedure, contributing to the overall success and efficiency of the cardiopulmonary bypass process [11].

#### Preoperatively haemodialysis

Within this study, three patients who had an estimated glomerular filtration rate (eGFR) below 45 mL/min were undergoing preoperative hemodialysis. These patients required additional renal support due to their compromised kidney function. The preoperative hemodialysis was performed through arteriovenous fistulas, which were pre-existing in these patients. By undergoing hemodialysis prior to the surgery, the aim was to optimize their renal status and ensure better management of their kidney function during the subsequent surgical procedure.

### Surgical procedure

A combination of inhaled and intravenous balanced anaesthesia, selected by the anaesthetist, was administered to the patients. The surgical procedure involved a traditional median sternotomy, followed by cannulation of the ascending aorta and right atrium using a biphasic cannula. Extracorporeal circulation was established using an adult membrane oxygenator, with a nasopharyngeal temperature maintained at 32 °C. Warm anterograde blood cardioplegia was utilized. Distal anastomoses were performed by clamping the aorta, and upon completion, the clamp was released. Subsequently, the aorta was laterally clamped to perform proximal anastomoses in the ascending aorta. Meanwhile, the blood was gradually warmed to reach the body's physiological temperature. For patients with severe impaired renal function (GFR < 60), during this warming process (approximately 30 min), ultrafiltration at a rate of 30–50 cc per minute was applied. After all anastomoses were completed and the patient was adequately warmed, and was weaned from the extracorporeal circulation.

### Graft conduits

All patients in the study underwent LIMA to LAD anastomosis. To achieve complete revascularization, the saphenous vein, obtained through open harvesting, was carefully prepared and anastomosed to the predetermined coronary branches.

### Data collection

Data for this study was collected from various reliable sources. Preoperative data were obtained by meticulously examining medical records from relevant departments, including cardiology, internal medicine, pulmonology, and nephrology. These records provided a comprehensive overview of variables such as age, BMI, gender, HT history, carotid artery disease, AMI, PMI, SHD, previous atrial fibrillation, T2DM, COPD, malignancy, anaemia, obesity, severe renal impairment, LVEF, eGFR, creatinine levels, HB, albumin, HCT, AST, ALT, HDL, LDL, and FEV1/FVC. Intraoperative data were meticulously collected by reviewing surgical records. This involved documenting critical information such as the number of distal anastomoses, cardiopulmonary bypass time, aortic cross-clamp time, instances of low cardiac output syndrome, prolonged bleeding, and any unfortunate occurrences of intraoperative death.

Postoperative data were gathered from both ICU and general ward records, in addition to consulting the national electronic health system records. This comprehensive approach allowed for the recording of vital variables including cardiopulmonary resuscitation, graft occlusion, stroke, bleeding, transfusion requirements (including FFP and PRBC), acute kidney injury, wound site infection, sternal dehiscence, Po HCT, Po Creatinine, intubation time, ICU duration, length of stay, and mortality. Furthermore, 12-month outcomes were meticulously evaluated using data extracted from the national electronic health system records. This analysis provided insights into significant factors such as the need for redo revascularization, renal replacement therapy, cardiac mortality, and all-cause mortality at the end of the 12-month follow-up period.

### Data analysis

The categorical data in this study were presented as percentages, which represent the proportion of individuals in each category. On the other hand, numerical data were presented as mean values and standard deviations (SD). To assess overall survival, Kaplan–Meier with the log-rank test was conducted for statistical analysis.

## Results

### Medical background

The study included individuals with a mean age of 60.7 ± 9.1 years and a mean BMI of 28.4 ± 4.1. The majority of participants were male (94.8%). Prevalence rates of medical conditions were observed, including HT (50.0%), CD (8.6%), AMI (3.4%), PMI (41.4%), SHD (6.9%), Previous AF (17.2%), T2DM (50.0%), COPD (27.6%), Malignancy (5.2%), Anaemia (41.4%), and Obesity (37.6%). Severe renal impairment was present in 20.6% of the participants. Various laboratory parameters were measured, including LVEF (54.6 ± 6.4), eGFR (74.8 ± 13.0), Creatinine (0.9 ± 0.19), HB (13.4 ± 1.6), Albumin (3.9 ± 0.5), HCT (41.1 ± 3.7), AST (25.5 ± 14.1), ALT (26.0 ± 12.6), HDL (40.9 ± 7.4), LDL (101.8 ± 37.6), and FEV1/FVC (73.3 ± 10.2). (Table 1).

**Table 1:** Medical background

**Medical Background**	**mean ± SD**	**% (n)**
Age (years)	60.7 ± 9.1	
BMI (kg/m²)	28.4 ± 4.1	
Gender (female)		5.2 (3)
HT		50.0 (29)
Carotid Artery Disease		8.6 (5)
AMI		3.4 (2)
PMI		41.4 (24)
SHD		6.9 (4)
Previous Atrial Fibrillation		17.2 (10)
T2DM		50.0 (29)
COPD		27.6 (16)
Malignancy		5.2 (3)
Anaemia		41.4 (24)
Obesity		37.6 (22)
Severe Renal Impairment		20.6 (12)
CRTT		5.2 (3)
LVEF (%)	54.6 ± 6.4	
eGFR (mL/min/1.73 m^2^)	74.8 ± 13.0	
Creatinine (mg/dL)	0.9 ± 0.19	
HB (g/dL)	13.4 ± 1.6	
Albumin (g/dL)	3.9 ± 0.5	
HCT (%)	41.1 ± 3.7	
AST (U/L)	25.5 ± 14.1	
ALT (U/L)	26.0 ± 12.6	
HDL (mg/dL)	40.9 ± 7.4	
LDL (mg/dL)	101.8 ± 37.6	
Fev1/FVC (%)	73.3 ± 10.2	

*AF* Atrial Fibrillation, *ALT* Alanine Aminotransferase, *AMI* Acute Myocardial Infarction, *AST* Aspartate Aminotransferase, *BMI* Body Mass Index, *CAD* Carotid Artery Disease, *CRRT* Continuous renal replacement therapy, *COPD* Chronic Obstructive Pulmonary Disease, *eGFR* Estimated Glomerular Filtration Rate, *FEV1/FVC* Forced Expiratory Volume in 1 s/Forced Vital Capacity, *HB* Haemoglobin, *HDL* High-Density Lipoprotein, *HCT* Haematocrit, *HT* Hypertension, *LDL* Low-Density Lipoprotein, *LVEF* Left Ventricular Ejection Fraction, *PMI* Perioperative Myocardial Infarction, *SHD* Structural Heart Disease, *T2DM* Type 2 Diabetes Mellitus

### Intraoperative outcomes

The intraoperative outcomes were as summarized in Table 2. the mean number of distal anastomoses was 3.3 ± 0.5. The cardiopulmonary bypass time was 87.5 ± 24.0 min, and the aortic cross-clamp time was 53.9 ± 19.3 min. The occurrence of low cardiac output syndrome was reported in 5.2% (*n* = 3) of cases, while prolonged bleeding and intraoperative death were not observed in any of the cases (0.0%).

**Table 2:** Intraoperative outcomes

**Intraoperative Outcomes**	**mean ± SD**	**% (n)**
Number of distal anastomosis	3.3 ± 0.5	
Cardiopulmonary Bypass Time (minutes)	87.5 ± 24.0	
Aortic Cross-Clamp Time (minutes)	53.9 ± 19.3	
Low Cardiac Output Syndrome		5.2 (3)
Prolonged Bleeding		0.0 (0)
Intraoperative Death		0.0 (0)

### Perioperative outcomes

The in-hospital outcomes observed in the study as summarized in Table 3. Cardiopulmonary resuscitation was required in 1.7% (*n* = 1) of cases. Graft occlusion occurred in 1.7% (*n* = 1) of cases. Stroke was reported in 3.4% (*n* = 2) of cases, while bleeding and transfusion requirement were observed in 3.4% (*n* = 2) of cases. Transfusions of FFP and PRBCs were required in 53.5% (*n* = 31) of cases. Acute kidney injury was present in 8.6% (*n* = 5) of cases, while wound site infection occurred in 13.8% (*n* = 8) of cases. Sternal dehiscence was reported in 5.2% (*n* = 3) of cases. The mean Po HCT was 30.9 ± 4.9, and the mean Po Creatinine was 1.2 ± 0.4. The mean intubation time was 8.9 ± 6.0 hours, and the mean ICU duration was 3.9 ± 1.2 days. The mean length of stay in the hospital was 9.6 ± 3.2 days. The overall mortality rate during the hospital stay was 3.4% (*n* = 2).

**Table 3:** In-hospital outcomes

**In-hospital Outcomes**	**mean ± SD**	**% (n)**
Cardiopulmonary resuscitation		1.7 (1)
MI		3.4 (2)
Graft Occlusion		1.7 (1)
Stroke		3.4 (2)
Bleeding		3.4 (2)
Transfusion requirement		53.5 (31)
Acute kidney injury		8.6 (5)
Wound site infection		13.8 (8)
Sternal dehiscence		5.2 (3)
Po HCT (%)	30.9 ± 4.9	
Po Creatinine (mg/dL)	1.2 ± 0.4	
Intubation Time (hours)	8.9 ± 6.0	
ICU duration (days)	3.9 ± 1.2	
Length of Stay (days)	9.6 ± 3.2	
Mortality		3.4 (2)

*ICU* Intensive care unit, *MI* Myocardial infarction, *Po HCT* Postoperative Hematocrit, *Po Creatinine* Postoperative Creatinine

### 12-month post discharge outcomes

**Fig. 1:**
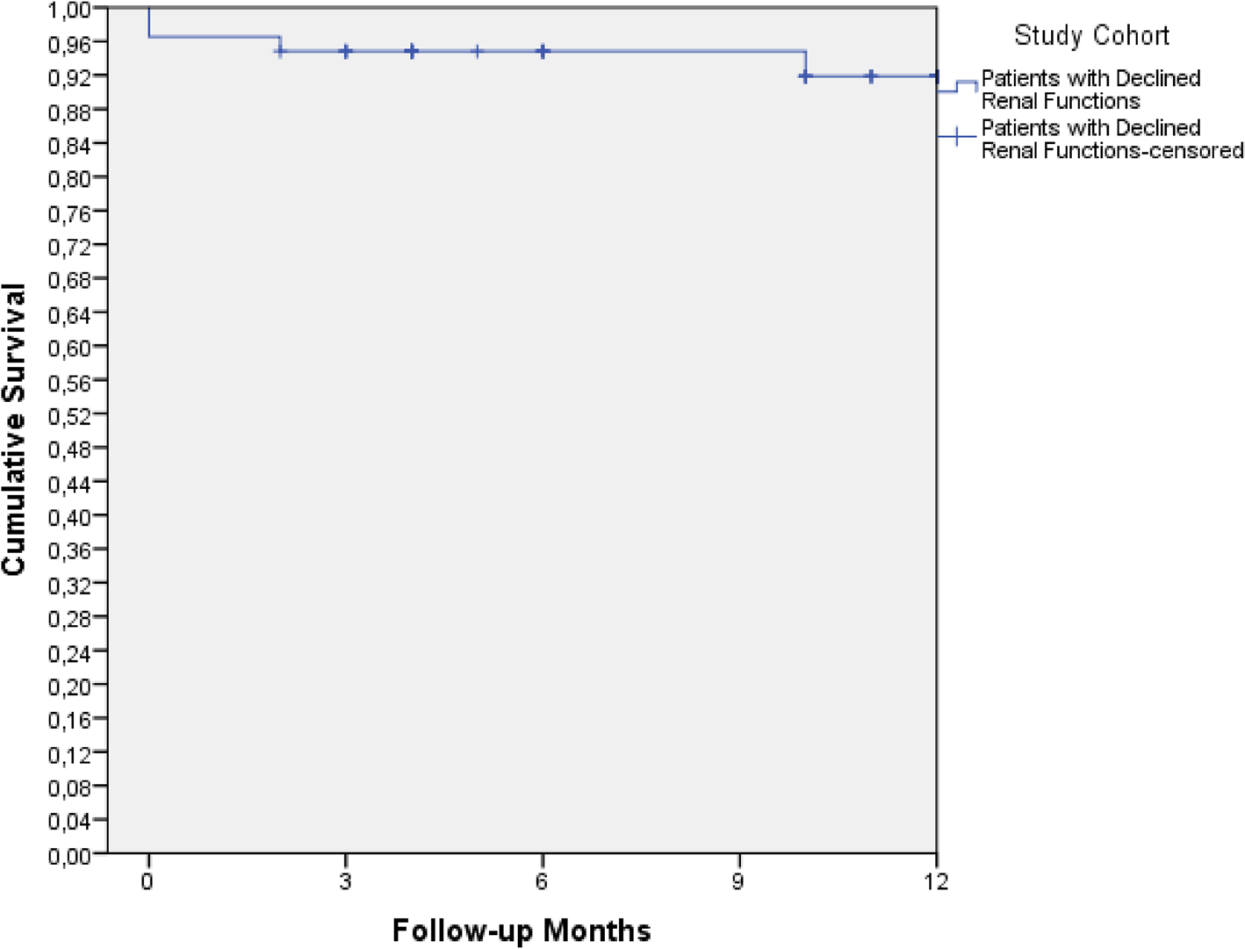
12-month overall survival

The 12-month post discharge outcomes observed in the study were as summarized in Table 4. there were no cases of redo revascularization or renal replacement therapy required within the first year after the procedure (0.0%, *n* = 0). The 12-month post discharge cardiac mortality rate was 1.7% (*n* = 1). The 12-month post discharge all-cause mortality rate was slightly higher at 3.6% (*n* = 2). To summarize, the 12-month survival rate stood at 93.1% (Fig. 1).

**Table 4:** 12-month post discharge outcomes

**12-Month Outcomes**	**% (n)**
12-month redo revascularization	0.0 (0)
12-month renal replacement therapy^a^	0.0 (0)
12-month cardiac mortality	1.7 (1)
12-month All-cause mortality	3.6 (2)

^a^Requirement of Continuous Renal Replacement Therapy (CRRT) in Patients Not on CRRT Preoperatively

## Discussion

Incomplete revascularization is defined as the limitation of revascularization to only large coronary arteries with a stenosis rate of more than 70 percent and/or a diameter of 2.5–3.5 mm, while neglecting to revascularize other diseased vessels. In such cases, plaques containing 50% stenosis and diseased arteries with a diameter of 2.0 mm or even 1.5 mm for PLV and PDA may persistently pose a risk of infarction in the future [7, 8].

On the other hand, a complete revascularization strategy involves the revascularization of all diseased vessels that are occluded more than 50% and have a diameter greater than 2.0 mm [8]. This comprehensive approach has garnered significant attention as it offers the promise of preventing potential risks associated with complications of plaque in the coronary arteries [7, 8].

The current literature suggests that complete revascularization offers overall survival advantages [12]. However, the feasibility of this approach in surgical patients, especially those with chronic kidney disease or poor renal function, is still debated.

According to the findings of our study, complications such as stroke, perioperative myocardial infarction, graft occlusion and bleeding in complete revascularization in patients with decreased renal function had comparable results with other revascularization methods described in the literature [2–6]. Patients who experienced perioperative morbidity were effectively managed and successfully discharged, with the exception of two unfortunate patients who passed away. Among those cases, one mortality was attributed to neurological causes, while another was attributed to cardiac reasons. Additionally, only one patient experienced graft occlusion; however, this individual underwent urgent percutaneous coronary intervention (PCI) and was safely discharged home. This finding not only highlights our successful management of graft occlusion but also supports the safety and efficacy of utilizing the saphenous graft for the right coronary artery in the context of complete revascularization.

On the other hand, the 12-month cardiac mortality rate and overall, 12-month survival rate were promising, while no need for re-intervention was observed at 12-month follow-up. Furthermore, our study indicated that post-operative acute kidney injury rates were comparable to those reported in the literature for patients with normal kidney function [13]. Importantly, none of our patients required renal replacement therapy in the post-operative period, implying that complete revascularization is safe for individuals with declining renal function.

### Strengths and limitations

The study on complete surgical revascularization in multivessel coronary artery patients with reduced renal function exhibits several strengths and limitations. One of its significant strengths is the comprehensive evaluation of a recommended treatment method, focusing on a specific patient group, which enhances the relevance and specificity of the findings. However, the study's limitations include a relatively small cohort, yielding a modest statistical power of 0.45 (45%) during post-hoc analysis compared to the closest study in the literature, cautioning against drawing definitive conclusions. Additionally, The study's single-centre and retrospective design introduce potential biases and may restrict the applicability of the findings to broader populations. The lack of a randomized controlled trial further reduces the ability to establish causality between complete revascularization and outcomes in patients with reduced renal function. Future research should address these limitations by conducting larger-scale studies with diverse cohorts and implementing randomized controlled trials.

## Conclusions

In conclusion, this study conducted a comprehensive evaluation of the outcomes of complete revascularization in patients with declined renal function. The results demonstrated that complete revascularization yielded complication rates comparable to those reported in the literature while presenting advantages during the 12-month follow-up period, such as reduced rates of re-intervention and postoperative cardiac mortality. These compelling findings highlight the potential benefits of complete revascularization for patients with declined renal function, underscoring the importance of conducting further randomized, and larger-sample sized research to optimize revascularization techniques specifically tailored to this patient population.

## Data Availability

The data that supports the findings of this study are available upon request. Due to the sensitive nature of the data and the confidentiality agreements in place, we are unable to publicly share the data directly. However, we are committed to promoting transparency in scientific research, and we encourage interested researchers to contact Corresponding Author to request access to the data.

## Abbreviations

AF: Atrial Fibrillation
AKI: Acute Kidney Injury
ALT: Alanine Aminotransferase
AMI: Acute Myocardial Infarction
AST: Aspartate Aminotransferase
BMI: Body Mass Index
CD: Carotid Disease
COPD: Chronic Obstructive Pulmonary Disease
CPR: Cardiopulmonary Resuscitation
eGFR: Estimated Glomerular Filtration Rate
FEV1/FVC: Forced Expiratory Volume in 1 s/Forced Vital Capacity
Hb: Hemoglobin
HCT: Hematocrit
HDL: High-Density Lipoprotein
HT: Hypertension
ICU: Intensive Care Unit
LDL: Low-Density Lipoprotein
LVEF: Left Ventricular Ejection Fraction
PMI: Post Myocardial Infarction
Po Creatinine: Postoperative Creatinine
Po HCT: Postoperative Hematocrit
SHD: Structural Heart Disease
T2DM: Type 2 Diabetes Mellitus
